# Changing environmental conditions and applying organic fertilizers in *Origanum vulgare* L.

**DOI:** 10.3389/fpls.2015.00549

**Published:** 2015-07-21

**Authors:** Bernardo Murillo-Amador, Luis E. Morales-Prado, Enrique Troyo-Diéguez, Miguel V. Córdoba-Matson, Luis G. Hernández-Montiel, Edgar O. Rueda-Puente, Alejandra Nieto-Garibay

**Affiliations:** ^1^Programa de Agricultura en Zonas Aridas, Centro de Investigaciones Biológicas del NoroesteS.C. La Paz, Mexico; ^2^Departamento de Agricultura y Ganaderia, Universidad de SonoraHermosillo, Mexico

**Keywords:** *Origanum vulgare*, production systems, bocashi, yield, water use efficiency

## Abstract

Any improvement in agricultural systems that results in higher production should also reduce negative environmental impacts and enhance sustainability. The aim of this research was to investigate the effect of two different production systems, one open-field and the other shade-enclosure with four bocashi doses, in order to find the best environmental option in terms of yield, physiological and morphometric characteristics in one oregano (*Origanum vulgare* L.) cultivar. In this study a completely randomized block design was used with four replications and evaluated for photosynthetic and transpiration rate, stomatal conductance, chlorophyll, leaf area and temperature, aerial and roots fresh and dry biomass, fresh and dry yield. The results showed that oregano adapted best to the shade-enclosure with increase yield of fresh and dry leaf weight of 165% and 118%, respectively, when compared to open-field. Also, higher doses of bocashi improved yield in both environments but more so in shade-enclosure. Soil moisture retention was higher in shade-enclosure which was reflected in physiological variables for soil matric potential, transpiration, stomatal conductivity, photosynthesis being significantly higher in shade-enclosure compared to open-field, thus improving yield. It seems that oregano plants can be grown and perform better under shade-enclosure than open-field and bocashi is a suitable organic fertilizer.

## Introduction

Herbal crops have been valued for centuries for their flavoring attributes and therapeutic properties (Kopsell et al., 2005). Oregano (*Origanum vulgare*, Lamiaceae) is an economically important herb producing secondary metabolites with functionally important properties that are useful for food industry applications. Oregano is one of the most important spices, it is a well-known medicinal and aromatic plant which grows throughout the Mediterranean area and in most parts of the Euro-Siberian region (Vokou et al., 1993) being *O. vulgare* ssp. *hirtum* (Link) Ietsw (syn.: *O. heracleoticum.*). Oregano is found all over the world, and is comprised of many species; it is well adapted to both dry and calcareous soils. Worldwide, *O. vulgare* is popular culinary herbal crops, and a variety of leaf products are produced from fresh or dry leaves, essential oils and seed markets. Oregano is of great economic importance but this is not only related to its use as a spice. Its essential oil has antimicrobial, cytotoxic, anti-oxidant and antifungal activity (Adam et al., 1998). It has been used as a remedy for respiratory disorders as an expectorant, for dyspepsia, painful menstruation as well as urinary tracts disorders. The active compounds include several essential oils, carvacrol, γ-terpinen, *p*-cymene, thymol, and myrcene, α-pinene; flavonoid naringenin and caffeic acid derivatives, in particular rosmarinic acid (PDR, 2004).

In Mexico, the state of Baja California Sur is the main producer of oregano; however, there is little or no research evaluating the local crops grown here (Murillo-Amador et al., 2013). This crop is mainly grown in open-field in an organic system where the plants are fertilized, irrigated and protected from pests and diseases throughout the growing period following standards accepted for cultivation of organic agriculture in this region (Murillo-Amador et al., 2006, 2010, 2013). Great attention has been paid toward the application of bio-organic farming to avoid the heavy use of agrochemical that has resulted in numerous environmental difficulties (Lampkin, 1999). The coincident application of organic manures and bio-fertilizers is frequently recommended for improving soil properties and obtaining clean agricultural products. In recent years, bio fertilizers effects have been studied mainly in open-field condition; however, little information is available on the response of herbs to different bio fertilizers doses under shade-enclosure conditions. In many countries there is great interest in changing agricultural management technologies based on eliminating the intensive use of agrochemicals. Nevertheless, chemical fertilizers have been in the past the key components for providing crop nutrients needs (Sharifi-Ashori-Abadi, 1998). But as a consequence, in many cases, using chemical fertilizers have caused negative environmental impacts such as soil, water and air pollution (Ghost and Bhat, 1998). In addition, these activities may also negatively influence the root biocoenosis by reducing beneficial microorganisms and mycorrhizal fungi (Plencette et al., 2005). However and due to the numerous concerns about a massive and indiscriminate mineral fertilization, the present tendency is to reduce significantly their use in agriculture (Van Noordwijk and Cadisch, 2002). Thus, future challenges will give priority on developing ecological and sustainable methods to alleviate their use to reduce negative impacts on crops in the 21st century (IPCC, 2007). Murty and Ladha (1988) and Mehnaz and Lazarovits (2006) mention the need for organic fertilizers. With respect to plant nutrition, organic matter can substitute, partially or totally, the use of mineral fertilizers (Bautista-Cruz et al., 2014). It is well accepted that for plant growth organic matter is needed (Varanini and Pinton, 1995). In most developing countries, the cost of mineral fertilizers is often limited to small-scale farms, since farmers are typically resource poor (Smestad et al., 2002). It is therefore imperative that other sustainable alternatives for soil fertility management are researched to ensure improved crop production and, consequently, improved food security. Organic fertilizers are one such alternative. One of the most promising organically based soil nutrient management practices is the use of bocashi, which uses a selected group of microorganisms to ferment organic waste. As compost, bocashi is used to improve the soil organic matter content. The addition of organic matter raises the chemical, physical and biological soil fertility. Bocashi is an organic fertilizer which is produced by different microorganisms from organic matter. The addition of bocashi to agricultural soils supplies nutrients and organic matter, decreases leaching of mineral elements from the soil and also improves soil physical structure. Bocashi has also been used to provide biological control against various plant pathogens (Hoitink and Grebus, 1994). Bocashi has already been established as a suitable fertilizer for improving the productivity of several medicinal and aromatic plants, such as *Dracocephalum moldavica* (Hussein et al., 2006), peppermint (O’Brien and Barker, 1996) and *Tagetes erecta* (Khalil et al., 2002).

On the other hand, it is well known that micro-climates were significantly different for the plants grown in shade-enclosure and open-field conditions. In this sense, it is important to study different microclimates that are generated when a shade-enclosure or open-field production systems is used, since other studies with other plant species have demonstrated that plants grown in shade-enclosure and open-field conditions have varying growth periods and responses, partly due to differences in the microclimates (Ganesan, 2002). In the open-field, most plants receive more sunlight than they can actually use for photosynthesis. Under shade-enclosure condition, light is one of the important microclimate factors that influence plant productivity apart from temperature, relative humidity and CO_2_ concentrations. Light has long been understood as the most important climate factor for plant growth (Boyer, 1982) and changes in irradiance will have an impact on plant growth, yield and morphology (Sionit et al., 1980; Dai et al., 2009; Craven et al., 2010), various aspects of physiology and cellular biochemistry (Cavagnaro and Trione, 2007; Favaretto et al., 2011), and plant productivity (Raveh et al., 1998). Previous results in many other plants have shown improved growth variables in shade-enclosure compared to open-field (Ganesan, 2002). Many researchers have shown that organic manures increase yield, growth and even essential oil production of celery (Mahajan et al., 1977), achilla (Sayed, 1993) groundnut (Mehta et al., 1995), roselle, cumin (Safwat and Badran, 2002), and fennel (Badran and Safwat, 2004). The hypothesis in this study is that shade-enclosure compared to open-field should improve the growth, yield, physiological and morphometric variables of oregano, and furthermore, that certain dosages of bocashi should have a beneficial effect independent of production system, although the degree may vary. Thereby, the objectives of current research was to assess the effects of two environmental conditions, shade-enclosure and open-field and four bocashi doses on chlorophyll, chlorophyll fluorescence, relative water content (RWC), leaf water potential (LWP), soil matric potential (SMP), transpiration, stomatal conductance, photosynthesis, water use efficiency (WUE), leaf temperature, fresh and dry plant yield, plant height, leaf area, aerial shoot length, fresh and dry weight, roots length, fresh and dry weight in oregano plants.

## Materials and Methods

### Ethics Statement

The research conducted herein did not involve measurements with humans or animals. The study site is not considered a protected area. No protected or endangered or species were used in the course of carrying out this study, thus no permissions were needed. However, to carry out research activities on the lands administered by Ejido Los Arados. Permission was granted by Mr. Francisco Higuera, the owner of the farm. The plant material used for this study was cultivated under shade conditions at Los Arados. The specie *O. vulgare* L. used in the present study is not considered an endangered species and their use therefore had negligible effects on broader ecosystem functioning.

### Study Area

The experiment growing oregano in two environments was carried out at Los Arados farm, located in the Peninsula of Baja California, Mexico (24°47′12.38″ N, 111°11′21.53″ W), 131 m.a.s.l. Mean, maximum and minimum temperatures in open-field were 25.3, 42.4, and 8.1°C with 65% of relative humidity and under shade-enclosure were 27.0, 48.5, and 6.3°C with 53% relative humidity during oregano cropping (August to December, 2013). The climatological conditions were captured with computerized weather station (Vantage Pro2^TM^ Davis Instruments, USA). García (1981) defined climate as semiarid Bw (h′) hw (e) with xerophytic vegetation. The soils are neutral to slightly alkaline at 20–60 cm depth, well aerated allowing for roots to penetrate with low organic matter content (less than 1%).

### Plant Material and Experimental Site Description

One regional cultivar of oregano was evaluated for physiological and morphometric characteristics in separate open-field and shade-enclosure environments. In May 1, 2013 seeds of oregano were sown into peat moss based medium in a germination tray for both environments. The seeds were cultured under shade conditions (35°C day/24°C night) during ∼30 days under natural photoperiods (lat. 24°47′ 12.38″ N). Seedlings were fertilized every 5 days with a Hoagland’s (Hoagland and Arnon, 1950) nutrient solution and watered as needed. 42,000 plants ha^-1^ were transplanted on June 3, 2013 into open-field and shade-enclosure conditions and land was prepared for transplanting according to the methodology described by Ruiz-Espinoza et al. (2010). Briefly, furrow spacing was 80 cm with a plant spacing of 30 cm. In the shade-enclosure, the model of the mesh was 1610 PME CR, this mean 16 × 10 threads cm^-2^ with holes of 0.4 × 0.8 mm, crystal color with 40% of shadow (monofilament stabilized polyethylene). Bocashi doses were randomized within plots, and plots were replicated four times in a randomized complete block design. Irrigation was applied in split applications such that plots received 3 and 4 mm water per week in shade-enclosure and open-field, respectively. Photosynthetically active radiation (PAR) over the growing period averaged 2398 and 2393 μmol m^-2^s^-1^ in open-field and shade-enclosure plots, respectively. The plants were adequate fertilized, irrigated and protected from pests and diseases throughout the growing period according the standard cultivation of organic agriculture of the region. No significant differences of the physico-chemical properties of soil between open-field and shade-enclosure were found. For shade-enclosure, the values were: pH 7.69, electrical conductivity 0.38 dS m^-1^, organic matter 0.61%, calcium 64.79 mg kg^-1^, magnesium 79.03 mg kg^-1^, sand 56.09%, silt 26.72%, clay 17.18%, carbonates 2.63 mg kg^-1^, bulk density 1.63 g cm^-3^, field capacity 22.85%, sol moisture 9.29%, wilting percent 13.56%, porosity 38.42%, nitrites 0.11 mg kg^-1^, nitrates 25.36 mg kg^-1^, phosphorus 1.18 mg kg^-1^ and sulfates 123.97 mg kg^-1^, while for open-field the values were: pH 7.71, electrical conductivity 0.32 dS m^-1^, organic matter 0.59%, calcium 67.49 mg kg^-1^, magnesium 72.93 mg kg^-1^, sand 58.92%, silt 25.64%, clay 15.18%, carbonates 2.99 mg kg^-1^, bulk density 1.66 g cm^-3^, field capacity 20.53%, soil moisture 8.53%, wilting percent 12.00%, porosity 33.30%, nitrites 0.10 mg kg^-1^, nitrates 24.79 mg kg^-1^, phosphorus 1.22 mg kg^-1^ and sulfates 112.58 mg kg^-1^.

### Bocashi Origin

The bocashi was processed at a location given by the following coordinates 24° 08’ 09.73″ N, 110° 25′ 41.73″ W, at 7 m.a.s.l., in the experimental field of Centro de Investigaciones Biológicas del Noroeste, S.C., located in La Paz, in the Peninsula of Baja California, Mexico. The chemical characteristics of the bocashi were an average of pH of 8.26, an electrical conductivity of 7.86 dS m^-1^, an organic matter content of 4.4%, calcium content of 601.2 mg kg^-1^, magnesium content of 121.56 mg kg^-1^, soluble phosphorus of 551.6 mg kg^-1^, total dissolved solids of 4.21 g L^-1^ and salinity of 4.3%.

### Photosynthesis, Transpiration and Stomatal Conductance

All variables were measured in three time periods (September 16, October 16, and November 15, 2013) on sunny days, which facilitated measurement under relatively stable conditions. Gas exchange measurements were performed for net CO_2_ assimilation rate hereafter referred as *A* (mol CO_2_ m^-2^ s^-1^), stomatal conductance hereafter referred as *Gs* (mol H_2_O m^-2^ s^-1^), and transpiration rate hereafter referred as *E* (mol H_2_O m^-2^ s^-1^) at 10:00–13:00 h (four replicates) in leaves as described by Ruiz-Espinoza et al. (2010), i.e., leaves that were similar in health, size and color. WUE hereafter referred as *WUE* was calculated as *A*/*E* (μmol CO_2_ μmol^-1^ H_2_O; Galmés et al., 2007). All measurements were carried out in open-field and shade-enclosure environments at saturating light (800 μmol m^-2^ s^-1^ over the photosynthetically active radiation waveband) using a portable infrared gas analyzer LCi Photosynthesis System (ADC^TM^ Bioscientific Ltd., England). During the measurements, climate conditions in the leaf chamber were set close to conditions in open-field or shade-enclosure, respectively, e.g., the CO_2_ concentration was 375.5 ± 7.2 and 363.3 ± 2.5 μmol mol^-2^ in shade-enclosure and open-field, respectively. The leaf temperature was set to 32 ± 1.5 and 36.8 ± 0.9°C with shade-enclosure and open-field, respectively. LWP hereafter referred as Ψ*_w_* (MPa) was measured in three time periods (September 16, October 16, and November 15, 2013) using a dew-point psychrometer (WP4-T, Decagon^TM^ Devices, Pullman, WA, USA) and in one plant per treatment, in mature leaves, which were exposed to direct light for at least 1 h before measurement, coinciding with the leaf gas exchange measurements. Soil matric potential hereafter referred as *SMP* (MPa), was measured in three time periods (September 16, October 16, and November 15, 2013), at the sample moment the soil surface was cleaned and the sample was extracted about 2 cm of depth, 1 cm close to the stem of the plant; the soil sample was introduced in a plastic tray of 4 cm of diameter per 1 cm of height, covered and introduced in an ice box and transported to the laboratory where *SMP* was measured using a dew-point psychrometer (WP4-T, Decagon^TM^ Devices, Pullman, WA, USA). Leaf temperature hereafter referred as *LT* (°C) was measured when the *A, Gs*, and *E* was determined using a portable infrared gas analyzer.

### Relative Water Content

Relative water content hereafter referred as *RWC* was measured in three time periods (September 16, October 16, and November 15, 2013) and average of the measures was obtained. Leaves were always collected from the mid-section, in order to minimize age effects. Following Ruiz-Espinoza et al. (2010) three disks were hole-punched from the same leaf (total area of 5.10 cm^2^). The disks were then immediately weighed (fresh mass, FM). In order to obtain the turgid mass (TM), disks were floated in distilled water inside a closed petri dish. During the imbibition period, leaf samples were weighed periodically, after gently wiping the water from the leaf surface with tissue paper. The petri dishes were maintained under dim light (around 20 μmol m^-2^ s^-1^) and under naturally fluctuating temperature conditions in the laboratory. At the end of the imbibition period, leaf samples were placed in a pre-heated 80°C oven (Shel-Lab^TM^, model FX-5, serie-1000203), and measured for constant weight (∼42 h), in order to obtain the dry mass (DM; Turner, 1979). All mass measurements were made using an analytical scale, with precision of 0.0001 g (Mettler^TM^ Toledo, model AG204). Values of FM, TM, and DM were used to calculate RWC, using the equation: RWC (%) = [(FM-DM)/(TM-DM)] × 100.

### SPAD-Readings

SPAD readings were taken in three time periods (September 20, October 21, and November 20, 2013) with a hand-held, dual-wavelength meter (SPAD 502, chlorophyll meter, Minolta^TM^ Camera Co., Ltd., Japan). Measurement procedure was carried out the same way according to MacNicol et al. (1976) and Ruiz-Espinoza et al. (2010) procedure was followed for averaging six SPAD readings per leaf (three per side) into a single number. A total of 300 leaves were measured, the same leaves were used for extractable chlorophyll and SPAD measurements.

### Chlorophyll a, b and Total

Chlorophyll was measured for three time periods (September 20, October 21, and November 20, 2013). Following SPAD measurement, Chlorophyll extraction and measurement procedure was carried out following procedure of Arnon (1949) and Ruiz-Espinoza et al. (2010) was followed for total chlorophyll. Briefly, chlorophyll is extracted with 80% acetone, kept in dark for 72 h and Chl *a* and Chl *b* absorbance measured at 645 and 663 nm, respectively in spectrophotometer (Spectronic Unicom^TM^, Cambridge, UK).

### Chlorophyll Fluorescence

Chlorophyll fluorescence parameters were measured in three time periods (September 20, October 21, and November 20, 2013), minimal fluorescence hereafter referred as *F_o_* with arbitrary units, maximal fluorescence hereafter referred as *F_m_* with arbitrary units and the ratio of variable fluorescence to maximal fluorescence with arbitrary units hereafter referred as *F_v_/F_m_* of youngest fully mature and expanded leaves (Reuter and Robinson, 1997) of two plants per treatment and replication were measured *in vivo* 30 min after darkness adaptation of the leaves with a set of dark adaption clips, which are small, and made from a lightweight plastic (Lichtenthaler and Burkart, 1999; Ruban and Johnson, 2009). The parameters were obtained using a field portable hand held chlorophyll fluorometer (OS-30p+ Opti-Sciences^TM^, Inc., Winn Avenue, Hudson, NH 03051, USA).

### Morphometric Measurements

Plant height (cm) was measured two times (September 5 and October 4, 2013) from the soil surface to the tip of the tallest flowering stem. This variable was measured during the growth period in three plants that were previously selected and labeled. During the last harvest (December 16, 2013) two plants per plot were randomly taken to measure the next variables, leaf area (cm^2^), which was determined with a Li-Cor (LI-3000A, Li-Cor^TM^, Lincoln, NE, USA). Aerial (shoots + leaves) fresh and dry weights (g); roots fresh and dry weights (g) were determined using an electronic balance (Mettler Toledo^TM^, Model PR2002, Switzerland); shoots and roots length (cm) were determined using a digital vernier (General No. 143, General Tools^TM^, Manufacturing Co., Inc. New York, NY, USA). Shoots, leaves and roots were oven-dried (Shel-Lab^TM^, model FX-5, serie-1000203) at 80°C until to constant weight was reached (∼48 h).

### Yield

Yield in fresh and dry weight (g plant^-1^) was determined at two harvesting time (on September 16 and December 16, 2013). The plants were collected in an area of 1.4 m^2^ just above the lignified parts of the stem, immediately weighed (yield fresh weight) in a digital balance (Scale^TM^, model 310136), then dried in the dark until it reached a constant weight and weighed (yield dry weight) in a digital balance (Scale^TM^, model 310136).

### Experimental Design and Statistical Analysis

Data were analyzed using univariate and multivariate analysis of variance (ANOVA and MANOVA) according to a factorial concept of two-way classification, being environments (open-field and shade-enclosure) and bocashi doses (0, 3, 6, and 9 t ha^-1^) modeled as fixed factors, with their interactions for a completely randomized block design with four replications. The difference between the means was determined by Tukey’s HSD multiple range test at *p* ≤ 0.05. In all cases, mean values were considered significantly different when *p* ≤ 0.05. RWC which is expressed in percentage, was arcsine transformed previous to ANOVA (Sokal and Rohlf, 1988). All analyses were done with Statistica software program v. 10.0 for Windows.

## Results

The MANOVA analysis did not show significant differences between environment (Wilks = 0.007, *F* = 5.83, *p* = 0.31), bocashi doses (Wilks = 0.00014, *F* = 0.93, *p* = 0.59) and the interaction environments × bocashi doses (Wilks = 0.000021, *F* = 1.91, *p* = 0.28). It can be seen that the relationship of Wilks possibilities was not significant. This means that few or no variables will show significant differences between the factors in the study when ANOVA is carried out by separating each variable independently (Johnson, 1988).

### Photosynthesis, Transpiration and Stomatal Conductance

Transpiration rate exhibited significant differences between environment (*F*_1,24_ = 27.53; *p* ≤ 0.0002) being lowest in open-field than shade-enclosure. Although *E* did not show significant differences between bocashi doses (*F*_3,24_ = 1.25; *p* ≥ 0.31) or between the interaction of environments × bocashi doses (*F*_3,24_ = 1.79; *p* ≥ 0.17), the *E* values were lowest in the doses of 0 t ha^-1^ and also in open-field following the same trend of increases as doses of bocashi increase in both environmental conditions (**Table 1**). Stomatal conductance displayed significant differences between environment (*F*_1,24_ = 43.75; *p* ≤ 0.0001), bocashi doses (*F*_3,24_ = 4.72; *p* ≤ 0.009) and the interaction of environments × bocashi doses (*F*_3,24_ = 3.47; *p* ≤ 0.03). The *Gs* values were lowest in open-field than shade-enclosure, being lowest at 0 t ha^-1^ and when the interaction was analyzed, the lowest values were in open-field at 0 t ha^-1^ displaying the trend of increase as bocashi doses increased in both environments (**Table 1**). Photosynthesis revealed significant differences between environment (F_1,24_ = 4.98; *p* ≤ 0.03) with higher values with shade-enclosure. Although photosynthesis did not display significant differences between bocashi doses (*F*_3,24_ = 0.87; *p* ≥ 0.47) or between the interaction of environments × bocashi doses (*F*_3,24_ = 0.85; *p* ≥ 0.47) the photosynthesis values were higher at the doses of 9 t ha^-1^ for both environments but higher for under shade-enclosure at 9 t ha^-1^. Photosynthesis proportionally in both conditions as bocashi doses increased (**Table 1**). No significant differences between environment (*F*_1,24_ = 0.06; *p* ≥ 0.81) were observed for *WUE*; however, the higher values were detected under shade-enclosure. This variable showed significant differences between bocashi doses (*F*_3,24_ = 4.22; *p* ≤ 0.01) being higher at 9 t ha^-1^ and increasing as bocashi doses increased. Not significant differences between the interaction of environments × bocashi doses (*F*_3,24_ = 1.84; *p* ≥ 0.16) were observed; nevertheless, *WUE* values increased as bocashi doses increased in both environmental conditions, with higher values at 9 t ha^-1^ in shade-enclosure (**Table 1**). No significant differences of *Ψ_w_* were exhibited between environment (*F*_1,24_ = 2.44; *p* ≥ 0.13), however, less negative values under shade-enclosure were observed. Also, this variable did not show significant differences between bocashi doses (*F*_3,24_ = 0.86; *p* ≥ 0.47) even though less negative values were observed as bocashi doses increased being less negative at 9 t ha^-1^. This variable showed significant differences between the interaction of environments × bocashi doses (*F*_3,24_ = 3.38; *p* ≤ 0.03) displaying less negative values in shade-enclosure at 9 t ha^-1^, while the more negative values was for dosages at 0 t ha^-1^ with open-field (**Table 1**). Significant differences between environment (*F*_1,24_ = 23.32; *p* ≤ 0.0006) were revealed for *SMP*, with less negative values at all bocashi doses with the shade-enclosure. This variable comparing the shade- with open field environment did not show significant differences between bocashi doses (*F*_3,24_ = 0.53; *p* ≥ 0.66) or between the interaction of environments × bocashi doses (*F*_3,24_ = 0.60; *p* ≥ 0.61), however, less negative values were exhibited as bocashi doses increased, displaying the same trend in the interaction of both factors with less negative values as bocashi doses increased in both environmental conditions (**Table 1**).

**Table 1:** Effect of environments **(A)**, bocashi doses **(B)** and the interaction of environments × bocashi doses **(C)** in the physiological characteristics in oregano grown in two environments (shade-enclosure and open-field) under four bocashi doses (0, 3, 6, and 9 t ha^-1^).

		Chl a (μg cm^2^)	Chl b (μg cm^2^)	Chl total (μg cm^2^)	SPAD readings	RWC (%)	LWP (MPa)	SMP (MPa)	E (mmol m^-2^ s^-1^)	Gs (mol m^-2^ s^-1^)	A (μmol m^-2^ s^-1^)	WUE (μmolC0^2^ μmol^-1^H_2_O)
**(A)**						
**Environments**						
Open-field		15.36 a	5.24 a	20.61 a	36.26 a	82.38 a	-2.89 a	-7.64 b	18.69 b	0.94 b	13.02 b	0.73 a
Shade-enclosure		16.65 a	5.62 a	22.28 a	35.76 a	82.52 a	-2.67 a	-0.37 a	24.40 a	2.15 a	17.34 a	0.78 a
**(B)**						
**Bocashi (t ha^-1^)**												
0		15.59 a	5.28 a	20.87 a	35.29 a	81.58 b	-2.92 a	-5.34 a	20.51 a	1.16 b	12.99 a	0.64 b
3		15.81 a	5.37 a	21.19 a	35.80 a	81.76 b	-2.87 a	-4.40 a	20.57 a	1.28 b	15.03 a	0.64 b
6		16.28 a	5.45 a	21.73 a	36.10 a	82.10 ab	-2.70 a	-3.44 a	22.13 a	1.72 ab	15.29 a	0.70 ab
9		16.36 a	5.63 a	22.00 a	36.85 a	84.37 a	-2.64 a	-2.83 a	22.99 a	2.02 a	17.40 a	1.03 a
**(C)**						
**Environments**	**Bocashi (t ha^-1^)**											
Open-field	0	14.94 a	5.11 a	20.06 a	34.66 a	81.41 a	-3.14 b	-10.34 a	17.75 a	0.70 c	10.96 a	0.61 a
Open-field	3	15.15 a	5.14 a	20.29 a	36.40 a	82.22 a	-3.04 b	-8.58 a	18.32 a	0.84 c	12.38 a	0.64 a
Open-field	6	15.38 a	5.26 a	20.65 a	36.85 a	82.74 a	-2.75 ab	-6.38 a	19.28 a	1.09 c	12.90 a	0.68 a
Open-field	9	15.99 a	5.46 a	21.45 a	37.14 a	83.16 a	-2.66ab	-5.24 a	19.42 a	1.13 c	15.85 a	1.00 a
Shade-enclosure	0	15.64 a	5.28 a	20.93 a	34.18 a	81.30 a	-3.10 b	-0.49 a	21.72 a	1.47 bc	13.09a	0.63 a
Shade-enclosure	3	15.79 a	5.29 a	21.09 a	35.36 a	81.46 a	-2.74 ab	-0.42 a	22.70 a	1.63 bc	17.68 a	0.67 a
Shade-enclosure	6	17.40 a	5.77 a	23.18 a	36.57 a	81.75 a	-2.59 ab	-0.34 a	26.50 a	2.60 ab	18.96 a	0.73 a
Shade-enclosure	9	17.79 a	6.15 a	23.93 a	36.95 a	85.59 a	-2.24 a	-0.22 a	26.70 a	2.92 a	19.63 a	1.06 a

*RWC, relative water content; LWP, leaf water potential; SMP, soil matric potential; E, transpiration; Gs, stomatal conductivity; A, photosynthesis; WUE, water use efficiency. Values within the same column with same letters are not significantly different at *p* < 0.05 (Tukey’s HSD multiple range test). The data corresponds to the average values from the three sampling periods (September 16, October 16, and November 15, 2013).*

### Relative Water Content

No significant differences between environment (*F*_1,24_ = 0.05; *p* ≥ 0.83) were found for this variable, displaying the same values in both environments, being just slightly higher in shade-enclosure (**Table 1**). The variable exhibited significant differences between bocashi doses (*F*_3,24_ = 4.01; *p* ≤ 0.01) being lower at 0 t ha^-1^ and increasing as bocashi doses increased. Despite not significant differences between the interaction of environments × bocashi doses were revealed (*F*_3,24_ = 1.67; *p* ≥ 0.19), the higher value was exhibited under shade-enclosure at 9 t ha^-1^, followed by open-field with a slightly trend to display higher values as bocashi doses increased in both environments (**Table 1**).

### Chlorophyll content

For chlorophyll *a* hereafter referred as *Chl a*, ANOVA did not show significant differences between environment (*F*_1,24_ = 2.63; *p* ≥ 0.11) displaying similar values in both environments being slightly higher in shade-enclosure. Neither this variable presented significant differences between bocashi doses (*F*_3,24_ = 0.218; *p* ≥ 0.88), though exhibited higher values at 9 t ha^-1^, increasing as bocashi doses increased. Even though this variable did not display significant differences between the interaction of environments × bocashi doses (*F*_3,24_ = 0.89; *p* ≥ 0.45) higher values were exhibited under shade-enclosure at bocashi doses of 9 and 6 t ha^-1^, followed by open-field at bocashi doses of 9 t ha^-1^ with a slightly trend to display highest values as bocashi doses increased in both environments (**Table 1**). For chlorophyll *b* hereafter referred as *Chl b*, ANOVA did not reveal significant differences between environment (*F*_1,24_ = 2.33; *p* ≥ 0.13), however, display analogous values in both environments being slightly higher in shade-enclosure. This variable neither shows significant differences between bocashi doses (*F*_3,24_ = 0.35; *p* ≥ 0.78) although the higher values were found at doses of 9 t ha^-1^, with increasing trend as bocashi doses increased. Despite these trends, this variable did not show significant differences between the interaction of environments × bocashi doses (*F*_3,24_ = 1.24; *p* ≥ 0.31) exhibited higher values under shade-enclosure at 9 and 6 t ha^-1^, followed by open-field at 9 t ha^-1^ with a slight increasing trend with higher values as bocashi doses increased in both environments (**Table 1**). In the same sense, total chlorophyll content hereafter referred as *total Chl* was not affected significantly by environments (*F*_1,24_ = 2.60; *p* ≥ 0.11), however, higher values were found under shade-enclosure. This variable neither shows significant differences between bocashi doses (*F*_3,24_ = 0.24; *p* ≥ 0.86) but displayed higher values at 9 t ha^-1^, increasing as bocashi doses increased. Notwithstanding the interaction environments × bocashi doses did not show significant differences (*F*_3,24_ = 0.98; *p* ≥ 0.41), this variable exhibited similar response to *Chl a* and *Chl b* with higher values under shade-enclosure at 9 and 6 t ha^-1^, followed by open-field at 9 t ha^-1^ with a slightly trend to exhibit highest values as bocashi doses increased in both environments (**Table 1**).

### SPAD Readings

SPAD readings did not exhibit significant differences between environments (*F*_1,24_ = 0.31; *p* ≥ 0.57) revealing similar values in both environments being slightly higher in open-field. Neither SPAD displayed significant differences between bocashi doses (*F*_3,24_ = 0.548; *p* ≥ 0.65), nevertheless showed a trend of increasing as bocashi doses increased with slightly higher values at 9 t ha^-1^. Despite the fact this variable did not reveal significant differences between the interaction of environments × bocashi doses (*F*_3,24_ = 1.24; *p* ≥ 0.31) the slightly upper values were exhibited under open-field at 9 t ha^-1^ followed by shade-enclosure at 9 t ha^-1^ with a little trend to display highest values as bocashi doses increased in both environments (**Table 1**). SPAD-502 readings were significantly related to total extracted chlorophyll. The best fit was a linear relationship. In terms of leaf area basis (μg cm^-2^), the correlation coefficient (*r*) value between SPAD-502 readings and total chlorophyll was positive and significant (*r* = 0.55, *p* ≤ 0.001, *N* = 32), indicating closer relationship of this variable with the SPAD-502 readings, i.e., higher the SPAD-502 readings higher will be the chlorophyll pigments and vice versa.

### Leaf Temperature

Leaf temperature exhibited significant differences between environments (*F*_1,24_ = 159.25; *p* ≤ 0.0001) showing higher values in open-field condition. Also, this variable revealed significant differences between bocashi doses (*F*_3,24_ = 5.41; *p* ≤ 0.005) displaying lower values at 0 t ha^-1^ and increasing lightly as bocashi doses increased. Although this variable did not show significant differences between the interaction of environments × bocashi doses (*F*_3,24_ = 0.61; *p* ≥ 0.61) the marginally upper values were exhibited under open-field with a slight trend to display highest values as bocashi doses increased in both environments (**Table 2**).

**Table 2:** Effect of environments **(A)**, bocashi doses **(B)** and the interaction of environments × bocashi doses **(C)** in the morphometric characteristics and leaf temperature in oregano grown in two environments (shade-enclosure and open-field) under four bocashi doses (0, 3, 6, and 9 t ha^-1^).

		Yield		Plant			Aerial		Roots			Leaf
		Fresh weight g plant^-1^	Dry weight g plant^-1^	Height (cm)	Leaf area (cm^2^)	Shoot length (cm)	Fresh weight (g)	Dry weight (g)	Length (cm)	Fresh weight (g)	Dry weight (g)	Temperature (°C)
**(A)**						
**Environments**						
Open-field		98.58 b	27.98 b	24.71 b	7441.13 b	32.18 b	189.56 b	45.88 a	34.09 a	227.95 b	91.88 b	36.80 a
Shade-enclosure		261.40 a	60.90 a	30.89 a	11349.81 a	39.81 a	437.74 a	102.52 a	35.40 a	446.84 a	137.59 a	31.97 b
**(B)**						
**Bocashi (t ha^-1^)**												
0		166.41 a	40.71 a	27.18 a	7034.76 a	32.43 a	296.07 a	68.26 a	34.12 a	288.23 a	97.26 a	33.14 b
3		167.33 a	41.26 a	27.29 a	8050.77 a	36.18 a	302.43 a	69.43 a	34.37 a	309.19 a	110.60 a	34.50 ab
6		192.45 a	47.70 a	28.05 a	10348.10 a	36.87 a	312.78 a	74.16 a	34.56 a	329.77 a	110.87 a	34.66 a
9		193.78 a	48.11 a	28.69 a	12148.25 a	38.50 a	343.31 a	84.96 a	35.93 a	422.38 a	140.22 a	35.25 a
**(C)**						
**Environments**	**Bocashi (t ha^-1^)**											
Open-field	0	64.11 b	18.58 d	21.28 a	5249.13 a	24.50 a	148.83 a	34.52 a	29.87 a	182.64 a	65.26 a	35.95 a
Open-field	3	79.07 b	22.44 cd	23.95 a	5589.25 a	32.25 a	182.47 a	43.66 a	30.25 a	193.96 a	75.43 a	36.75 a
Open-field	6	103.91 b	29.81 cd	26.80 a	7806.38 a	33.75 a	195.91 a	46.45 a	37.00 a	225.93 a	98.03 a	36.93 a
Open-field	9	147.24 b	41.10 bcd	26.83 a	11119.78 a	38.25 a	231.01 a	58.91 a	38.87 a	309.26 a	128.81 a	37.57 a
Shade-enclosure	0	228.91 a	52.71 ab	29.30 a	8820.40 a	38.75 a	394.56 a	89.42 a	30.25 a	392.45 a	123.72 a	30.33 a
Shade-enclosure	3	237.65 a	54.30 ab	30.42 a	10512.30 a	40.00 a	409.67 a	92.85 a	34.87 a	393.83 a	129.26 a	32.07 a
Shade-enclosure	6	270.54 a	62.83 ab	30.55 a	12889.83 a	40.12 a	456.02 a	104.34 a	38.00 a	465.59 a	145.76 a	32.56 a
Shade-enclosure	9	308.49 a	73.78 a	33.30 a	13176.73 a	40.37 a	490.71 a	123.47 a	38.87 a	535.50 a	151.63 a	32.92 a

*Values within the same column with same letters are not significantly different at *p* < 0.05 (Tukey’s HSD multiple range test).*

### Chlorophyll Fluorescence

**FIGURE 1:**
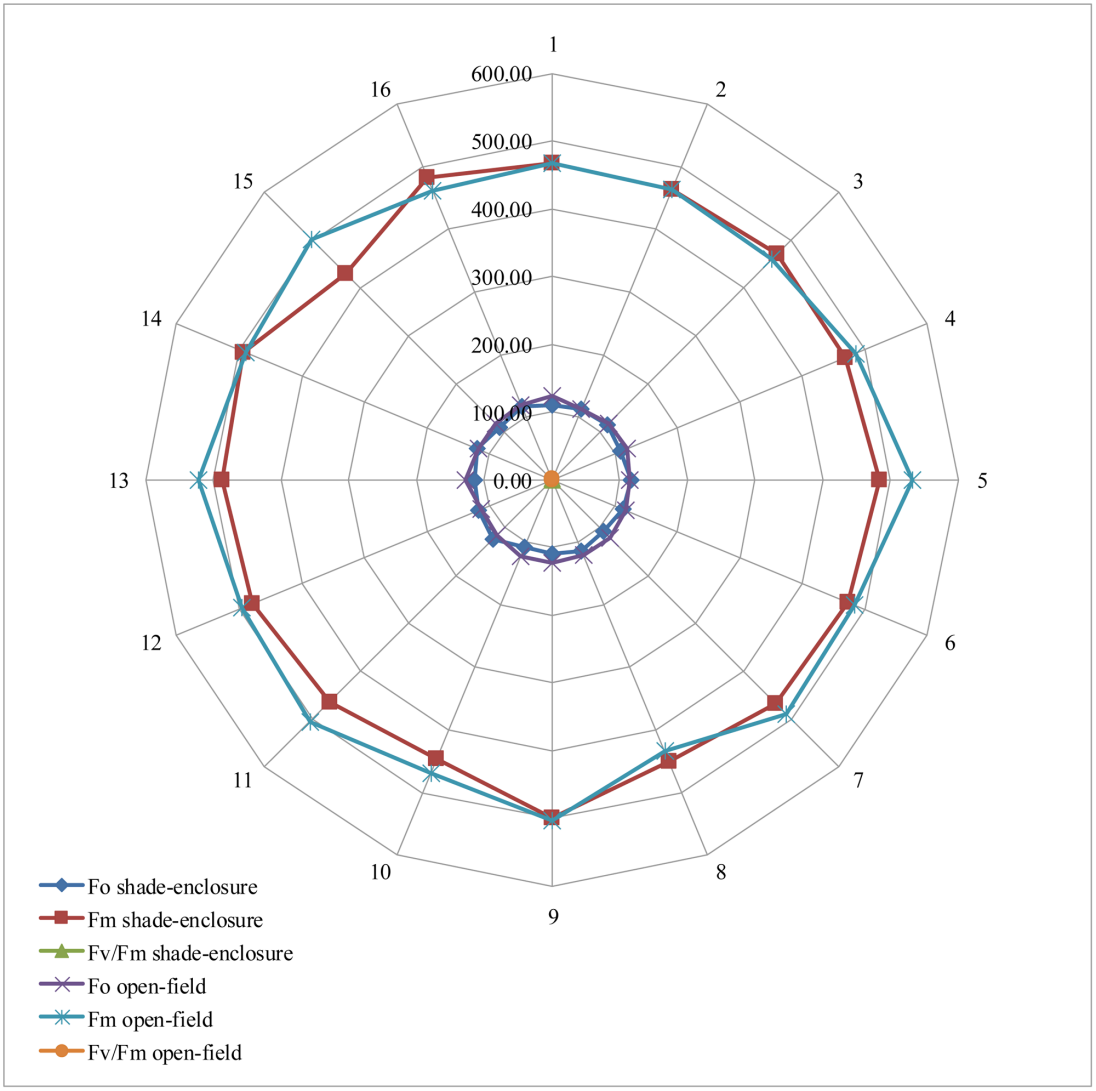
**Effect of the interaction of environments × bocashi doses in the chlorophyll fluorescence of oregano grown under shade-enclosure and open field and four bocashi doses (0, 3, 6, and 9 t ha^-1^).** Data are mean, *n* = 4.

Significant differences in *F_o_* were found between environment (*F*_1,24_ = 10.60; *p* ≤ 0.003) showing higher values (119.13 ± 4.04) in open-field condition than shade-enclosure (113.73 ± 4.38; **Figure 1**), while not after were significant differences were found between bocashi doses (*F*_3,24_ = 0.54; *p* ≥ 0.65) or between the interaction of environments × bocashi doses (*F*_3,24_ = 0.08; *p* ≥ 0.96), however, *F_o_* showed slightly higher values at 9 t ha^-1^ in both conditions, 115.17 ± 3.24 and 120.75 ± 4.23 with the shade-enclosure and open-field, respectively. Also, this variable showed a trend to increase marginally as bocashi doses increased. The parameter *F_m_* did not show significant differences between environment (*F*_1,24_ = 3.41; *p* ≥ 0.07), bocashi doses (*F*_3,24_ = 0.68; *p* ≥ 0.57) or between the interaction of environments × bocashi doses (*F*_3,24_ = 0.40; *p* ≥ 0.75), however, the higher values were found in those plants under open-field (485.17 ± 24.25) than shade-enclosure (470.48 ± 16.95) condition (**Figure 1**). Also, this variable, showed a trend to increase a little bit as bocashi doses increased. The quantum yield of PSII, as indicated by *F_v_/F_m_* in the dark, was not affected by the environment treatments (*F*_1,24_ = 0.84; *p* ≥ 0.36), bocashi doses (*F*_3,24_ = 0.99; *p* ≥ 0.41) or between the interaction of environments × bocashi doses (*F*_3,24_ = 0.30; *p* ≥ 0.82). This variable showed an average of 0.75 ± 0.01 with the shade enclosure and 0.76 ± 0.02 with open-field, respectively. Also, this variable showed a trend to increase a bit as bocashi doses increased, showing an equal value of 0.76 at 9 t ha^-1^ in both conditions (**Figure 1**).

### Yield

Yield in fresh weight showed significant differences between environment (*F*_1,24_ = 86.83; *p* ≤ 0.0001) showing higher values in shade-enclosure condition. This variable did not show significant differences between bocashi doses (*F*_3,24_ = 0.75; *p* ≥ 0.053); however, display lower values at 0 t ha^-1^ and increasing lightly as bocashi doses increased. This variable exhibited significant differences between the interaction of environments × bocashi doses (*F*_3,24_ = 3.55; *p* ≤ 0.02) displaying higher values under shade-enclosure with a trend to display highest values as bocashi doses increased in both environments (**Table 2**). Yield in dry weight showed similar results than yield in fresh weight, with differences between environment (*F*_1,24_ = 46.82; *p* ≤ 0.0001) with higher values in shade-enclosure condition. In the same sense, this variable did not show significant differences between bocashi doses (*F*_3,24_ = 0.75; *p* ≥ 0.053); nevertheless, display lower values at 0 t ha^-1^ and increasing lightly as bocashi doses increased. Also, this variable demonstrated significant differences between the interaction of environments × bocashi doses (*F*_3,24_ = 3.44; *p* ≤ 0.03) exhibiting higher values under shade-enclosure with a trend to display highest values as bocashi doses increased in both environments (**Table 2**).

### Morphometric Measurements

Significant differences between environment (*F*_1,24_ = 23.45; *p* ≤ 0.0006) were observed for plant height, with higher values under shade-enclosure. Although this variable did not show significant differences between bocashi doses (*F*_3,24_ = 0.30; *p* ≥ 0.82) and between the interaction of environments × bocashi doses (*F*_3,24_ = 2.75; *p* ≥ 0.06), the lower values were at 0 t ha^-1^ and increased as bocashi doses increased, showing a clear trend with higher values under shade-enclosure at higher bocashi doses and increasing under open-field and increasing as bocashi doses increased (**Table 2**). Similar to plant height, the variable shoot length showed significant differences between environment (*F*_1,24_ = 7.31; *p* ≤ 0.01) being higher with the shade-enclosure, but no significant differences were observed between bocashi doses (*F*_3,24_ = 0.82; *p* ≥ 0.49) and between the interaction of environments × bocashi doses (*F*_3,24_ = 1.26; *p* ≥ 0.30), nonetheless, the trend of this variable shows higher values as bocashi doses increased and in the same sense, showed higher values under shade-enclosure at higher bocashi doses and increased under open-field and as bocashi doses increased (**Table 2**). Significant differences between environment (*F*_1,24_ = 34.30; *p* ≤ 0.000) were observed for aerial (shoots + leaves) fresh weight with greater values under shade-enclosure. This variable did not show significant differences between bocashi doses (*F*_3,24_ = 0.24; *p* ≥ 0.86) or between the interaction of environments × bocashi doses (*F*_3,24_ = 0.61; *p* ≥ 0.61), in spite of this, greater values were exposed at higher bocashi doses and when the interaction was take into account, the upper values were under shade-enclosure with higher bocashi doses in both conditions (**Table 2**). Aerial dry weight showed similar response that fresh weight, with significant differences between environment (*F*_1,24_ = 35.05; *p* ≤ 0.0004) with bigger values under shade-enclosure, but did not show significant differences between bocashi doses (*F*_3,24_ = 0.63; *p* ≥ 0.60) or between the interaction of environments × bocashi doses (*F*_3,24_ = 1.20; *p* ≥ 0.32), then again, the greater values were at higher bocashi doses and in the interaction were higher under shade-enclosure and decreased in both conditions as bocashi doses decreased (**Table 2**). Leaf area showed significant differences between environment (*F*_1,24_ = 6.13; *p* ≤ 0.02) with considerably higher values under shade-enclosure. This variable did not show significant differences between bocashi doses (*F*_3,24_ = 2.12; *p* ≥ 0.12) or between the interaction of environments × bocashi doses (*F*_3,24_ = 0.19; *p* ≥ 0.89), though, the response was in the same sense than other variables, showing higher values as bocashi doses increased, and when the interaction was analyzed, the higher values were observed under shade-enclosure at 9 and 6 t h^-1^ following a defined pattern of increased as bocashi doses increased in both conditions (**Table 2**). Roots length did not show significant differences between environment (*F*_1,24_ = 0.20; *p* ≥ 0.65), bocashi doses (*F*_3,24_ = 0.07; *p* ≥ 0.97) or between the interaction of environments × bocashi doses (*F*_3,24_ = 2.08; *p* ≥ 0.12), but slightly higher values were found under shade-enclosure, and these values increased as bocashi doses increased, while the interaction showed a particular response because of higher values were found in both conditions at 9 t ha^-1^, following a definite increasing trend with doses (**Table 2**). Roots fresh weight exposed significant differences between environment (*F*_1,24_ = 16.55; *p* ≤ 0.0004) with greater values under shade-enclosure, but this variable did not show significant differences between bocashi doses (*F*_3,24_ = 1.20; *p* ≥ 0.32) or between the interaction of environments × bocashi doses (*F*_3,24_ = 0.16; *p* ≥ 0.92), although a clear trend was displayed with respect to bocashi doses, because root fresh weight increased as bocashi doses increased. Also, the results showed higher values of this variable under shade-enclosure and increased as bocashi doses increased in both conditions (**Table 2**). Roots dry weight showed similar response to root fresh weight, with significant differences between environment (*F*_1,24_ = 4.35; *p* ≤ 0.04) with superior values under shade-enclosure. Although this variable did not exhibited significant differences between bocashi doses (*F*_3,24_ = 0.68; *p* ≥ 0.56) or between the interaction of environments × bocashi doses (*F*_3,24_ = 0.32; *p* ≥ 0.80), the trend was similar for other morphometric variables with higher values occurring with larger bocashi doses and under shade-enclosure and increasing as bocashi doses increased (**Table 2**).

## Discussion

Here we have documented the effects of two production systems, open-field and shade-enclosure, and four bocashi doses (0, 3, 6, and 9 t ha^-1^), to find the best environmental option to physiological, morphometric characteristics and yield in oregano. The results showed that oregano had 165 and 118%, greater fresh and dry leaf yield in the shade-enclosure compared to open-field. This significant yield improvement was undoubtedly due to better soil moisture retention in shade-enclosure which was reflected in physiological variables for *SMP*, transpiration (*E*), stomatal conductivity (*Gs*), photosynthesis (*A*) being significantly higher in shade-enclosure. Although other physiological parameters such as chlorophyll content, *RWC LWP* and *WUE* were not significantly different in both environments, they were nevertheless higher values in the shade-enclosure.

*Gs E* and *A* of oregano plants grown under open-field decreased indicating that WUE of the plants grown in open-field decreased. While in contrast, the metabolism of oregano plants grown under shade-enclosure conditions is benefited by increased *E, Gs*, and *A* which is the basis of the increase of yield (Pessarakli, 2005). Leaf photosynthesis is the component of canopy photosynthesis that is known to account for most of the variation in yield (Takai et al., 2010). The improvement in *E, Gs*, and *A* in shade-enclosure environment is due to higher soil moisture retention and was reflected by significantly higher (more positive *SMP* allows for higher water content) in *SMP* (-0.37 MPa) in shade-enclosure compared to open-field (-7.64 MPa). A higher more positive *SMP* value indicates higher water content in soil is available for osmotic plant use in the shade-enclosure, since SMP variation depend of the soil physical properties.

Recently, Murillo-Amador et al. (2013) found that for oregano and thyme species, all variables measured under shade-enclosure conditions were more favorable than open-field condition. The *SMP* under shade-enclosure, showed less negative values than open-field condition, probably because the soil under shade-enclosure condition dry more slowly as water is retained, and in addition this is related with the higher *Gs, E*, and *A* in oregano plants grown under this condition but with similar *WU*E than open-field, maintaining less negative *LWP* under shade-enclosure and therefore slightly higher *RWC* values in those plants grown under shade-enclosure. These results could also possibly be related with higher CO_2_ content in shade-enclosure. Other studies has been suggested that plants with high CO_2_ availability use less water (Vu et al., 1998), have lower osmotic pressure (Tyree and Alexander, 1993), and have better growth (Vu et al., 1998). This contrasts with other studies which indicate high CO_2_ causes lower transpiration and stomatal conductance (Bunce, 1998). In our case, the opposite condition occurred in shade-enclosure transpiration and stomatal conductance increased and as a consequence yields of fresh and dry leaf weight increased.

In the present study, the CO_2_ concentration was 375.5 ± 7.2 and 363.3 ± 2.5 μmol mol^-2^ under the shade-enclosure and open-field, respectively. Oregano cultivated today is still a wild species (i.e., not completely domesticated) that has been exposed to the elements, therefore it is considered highly resistant to drought, which was demonstrated in the present study when those plants showed higher rates of *E* in both conditions, but contrary to the what was expected, resulted with not significant differences in WUE between environments and consequently with similar RWC values in both environmental conditions and maintaining less negative *Ψ*w under shade-enclosure despite of the higher *E* and *Gs*. In other species, such as barley, decreases in *RWC* have been related to higher rates of transpiration (Robredo et al., 2007). In addition, Teulat et al. (1998) reported in barley that *RWC* decreased as a result of soil drying.

However, oregano seems to be a more drought-resistant species since even when soil in the water is unlimited or saturated such as in shade-enclosure, we observed increase in *LWP*, despite the fact that both *Gs* and *E* increased under shade-enclosure. Definitively, the *E, Gs*, and *A* in oregano plants, were controlled by the environmental conditions of growth since significant differences between environments were found; however, these physiological variables are controlled not only by external physical factors but also by their specific physiology (García-Quijano and Barros, 2005). Numerical differences in *LWP* of oregano plants in both shade-enclosure and open-field conditions may have been due to differences in soil water or nutrient content.

As expected, *LT* was higher in those oregano plants grown under open-field and as bocashi doses increased, however, this increase in *LT* not affected other physiological variables such as *Gs, E*, and *A*, despite of have been demonstrated that *LT* determines the carboxylation velocity by affecting the electron transport rate per unit of chlorophyll, the amount of nitrogen in rubisco and the turnover number of carboxylation sites (García-Quijano and Barros, 2005). Since water deficit has multiple effects in plants, including reductions in *Gs*, carboxylation efficiency and PSII activity (Lawlor and Tezara, 2009), all of which affects photosynthetic capacity, *WUE* and productivity in general. In the present study, the use of use shade-enclosure as a production system in arid areas, represent an advantage to the plants since the environments conditions change around the plants modifying the soil humidity, increasing the water availability for the plants, and then, increasing or improvement the physiological, morphometric characteristics and yield. The consequent protracted stomata aperture contributes to the higher photosynthetic and transpiration rates. However, the improved water status of plants might be due, at least partially, to the effects of shade-enclosure condition, since the physiological and morphometric characteristics and yield are influenced not only by the genetic also by environmental conditions which can result in biochemical and physiological alterations in plant (Sangwan et al., 2001). Definitively, the use of bocashi as organic fertilizer is not only the organic materials, manures and residual of plants but also as bacteria and fungi, especially plant growth promoting bacteria (PGPB), which release phytohormones, then, bocashi get better growth and development of oregano plants and improving all physiological variables. Also, biofertilizers such as bocashi has a high porosity and a high ability for absorption and conservation of mineral nutrients, and is enriched with several beneficial soil microbes and also contains many essential plant nutrients like N, P, and K. Besides, the organic matter decreases leaching of mineral elements from the soil and also improves soil physical structure, improving root development, providing plant nutrients and enhancing nutrient uptake by plants (Hussein et al., 2006). Varanini and Pinton (1995) has pointed out the importance of soil organic matter for providing water to the plant at the correct pH and as a platform for beneficial micro-organisms for root development. Bocashi facilitates water absorption and retention by the soil, which has a favorable effect on growth, then, all minerals were available for oregano plants, improving physiological and growth variables. For microorganisms, organic matter content in soils is a potential nutrient supply (Milpa-Mejía et al., 2012). Hussein et al. (2006) demonstrated that organic matters such as compost can improve soil structure, improving root development, providing plant nutrients and enhancing nutrient uptake by plants. Moreover, compost facilitates water absorption and retention by the soil, which has a favorable effect on growth and essential oil components of plants. Biofertilizers has already been established as a suitable fertilizer for improving the productivity of several medicinal and aromatic plants, such as *Dracocephalum moldavica* (Hussein et al., 2006), peppermint (O’Brien and Barker, 1996), and *Tagetes erecta* (Khalil et al., 2002). The reported promoting effect of biofertilizers on the growth and physiological characteristics of aromatic plants such as oregano may be due to their ability to enhance the physical, chemical, and biological properties of the soil. Furthermore, this may be related to the good balance of nutrients and water in the root zone (Abdelaziz et al., 2007; Gharib et al., 2008; Darzi et al., 2009). The increases of values of all physiological and morphometric variables including yield as bocashi doses increased might be related to the helpful effect of bocashi in increasing the root surface area per unit of soil volume, water-use efficiency and photosynthetic activity. Darzi et al. (2009) reported improved plant essential oils quality in sweet fennel using compost and vermicompost which might be related to the positive effect of these fertilizers in increasing the root surface area per unit of soil volume, photosynthesis and water-use efficiency (Abdelaziz et al., 2007).

Another fundamental variable measured in the present study was chlorophyll fluorescence; however, their parameters (*F_o_, F_m_*, and *F_v_/F_m_*) did not show significant differences between environments (except *F_o_* for environments), bocashi doses and their interaction. As expected, the chlorophyll fluorescence parameters showed slightly higher values with open-field, as have been demonstrated that these parameters will be slightly higher on sun leaves than on shade leaves (Lichtenthaler and Babani, 2004). In the present study, all parameters increased as bocashi doses increased in both environments. Although this study not included biotic or abiotic stress as main factors, through the chlorophyll fluorescence parameter using the ratio *F_v_/F_m_* (Baker and Oxborough, 2004) it is possible to see signs of stress in plants much earlier than with traditional phenotypic methods. In recent years, the analysis of chlorophyll fluorescence has become ubiquitous in plant ecophysiology studies, and the principle underlying this technique is relatively straightforward. Light energy absorbed by chlorophyll molecules can (1) be used to drive photosynthesis, or (2) be dissipated as heat or (3) be re-emitted as light -chlorophyll fluorescence- (Oxborough, 2004). Under optimal growth conditions much of the energy absorbed from light is directed into photosynthesis and plants emit a basal level of chlorophyll fluorescence. However, when the photosynthesis apparatus is under abiotic stress, the balance is disturbed and more energy must be released through heat and fluorescence (Müller et al., 2001). According to Maxwell and Johnson (2000), maximum *F_v_/F_m_* values vary with species. The optimal *F_v_/F_m_* reading for stress free plants is in the range of 0.79–0.84. In the present study, the average was 0.75 for plants under shade-enclosure and 0.76 for plants under open-field. The ratio *F_v_/F_m_* offers a number of advantages since values have been found to correlate to carbon assimilation in regards to plant stress types that affect PSII; samples are dark adapted to the same known state for comparison purposes, and it is the most used chlorophyll fluorescent parameter in the world. We are in agreement with Lichtenthaler and Babani (2004) that field plants should only be compared to field plants and greenhouse plants should be compared to greenhouse plants due to light history. Also, it is important to compare samples of similar light history, due to the fact that it can take up to 60 h for relaxation of chronic photo inhibition (Lichtenthaler and Babani, 2004) which will affect plant yield. However, in this study a comparison was necessary of the two environmental systems in order to determine which improves yield. Luckily, effect of light history was found to have no effect, since it was found that *F_v_/F_m_* was not significantly different in both systems or with bocashi doses. This despite the fact that it is known that *F_v_/F_m_* is affected by both photochemical and non-photochemical factors, showing a decline in *F_v_/F_m_* due to a decrease in reaction centers capable of photochemistry or reversible non-photochemical quenching (Baker and Oxborough, 2004). This indicates that oregano plants that absorbed light energy by chlorophyll molecules received similar quantity of light in both growth conditions including bocashi doses.

Leaf chlorophyll has been used to quantify photosynthetic health of plants (Wittmann et al., 2001). Decreases in chlorophyll content in open-field environment rather than shade-enclosure indicate that light intensity was greater in open field. Jason et al. (2004) reported that decreases in *Chl b* content is an indicator of chlorophyll destruction by excess irradiance (Jason et al., 2004). Plants grown under shade-enclosure condition are known to increase chlorophyll density to capture more light energy (Wittmann et al., 2001). The increase in leaf chlorophyll content in shade-enclosure demonstrated the oregano plants ability to improve light energy capture. This has been reported by others (Kura-Hotta et al., 1987; Lei et al., 1996). *Chl b* is usually the main component of light harvesting chlorophyll protein, so that the amount of this pigment is important for the capacity of the leaf to accommodate to the shade-enclosure condition, whereas *Chl a* is concentrated around PSII. To capture as much light as possible, shade grown plants typically have more light-harvesting complexes per unit area than sun-grown plants. Smaller leaves found in oregano grown under open-field probably result in a thicker mesophyll cell layer of the leaves (Bosabalidis and Kokkini, 1997). Mesophyll cells contain the chloroplasts which capture light energy for the photosynthesis process. The chloroplasts contain an internal membrane called thylakoid membrane composed of pigments (especially chlorophyll). For this reason it can be suggested that thicker mesophyll could be the reason for the lower chlorophyll content which was found in oregano leaves grown under open-field. Chlorophyll content of oregano plants was the highest under shade-enclosure, whereas that those plants under open-field was the lowest, indicating that the chlorophyll content might be contributed to the difference in the photosynthetic capacity among the environmental conditions. The role of pigments in plant processes is indispensable, whereas the synthesis of photosynthetic pigments is genetically controlled, but it also depends on environmental factors. Fresh and dried oregano herb is widely used in flavorings, herbal tea and food preparations. Thus, it is important to know its yield in fresh and dry weight. In the present study, oregano showed the maximum yield of fresh and dry weight under shade-enclosure. Similar results were found by Murillo-Amador et al. (2013) in oregano and thyme. In the present study, leaf area reached maximum values when plants were grown under shade-enclosure environment (**Table 2**). These results are different than those found by Gordon et al. (1994) in *Posidonia sinuosa* where they reported in reduced light a decrease in the size of leaves. According to Campbell and Miller (2002), oregano plants could be do an adjustment which increase the respiratory demand of the shoot to help compensate for the greatly decreased the photosynthetic capacity of the leaves. The fact that all morphometric variables measured showed higher values at plants under shade-enclosure environment, suggest that oregano, could be considered as shade-tolerant species, since it was shown to acclimatize well under shade-enclosure conditions.

This could possibly be due to a lower photosynthetic respiration rate (Yeh, 1996). The increase of morphometric characteristics of oregano plants as bocashi doses increased undoubtedly is due to the beneficial effect of the organic manure. This has been shown with plant height by Abdou and Mahmoud (2003), and Mohamed and Ahmed (2003). Specific improvements in morphometric characteristics with bocashi have been reported for various plants. Badran and Safwat (2004) with fennel plant, Swaefy et al. (2007) with peppermint plant, Abou-Aly and Gomaa (2002) with coriander plant, and Shaalan (2005) with *Nigella sativa* L. plant.

## Conclusion

The main highlight of this study showed that oregano adapted best to the shade-enclosure compared to open-field providing improvement in fresh and dry leaf weight yield of 165 and 118%, respectively. This yield improvement was not caused by differences in light environment, since none was found, but in improved SMP in shade-enclosure facilitating osmotic intake in plants. This was reflected in significantly better physiological variables of transpiration, stomatal conductance, photosynthesis and as mentioned previously in SMP with plants in the shade-enclosure, thus resulting in significantly better plant yield. Not only were yield of fresh and dry weight greater in shade-environment, but morphometric characteristics such as plant height, leaf area, aerial shoot length, roots length, achieved better results compared to open-field. Although chlorophyll, RWC, leaf water content, and WUE were not statistically significantly between the two environments, nevertheless these variables performed better under the shade-enclosure. In addition, this research revealed that the application of increasing doses of bocashi, significantly improve physiological variables such as chlorophyll, RWC, leaf water content, SMP, transpiration, stomatal conductance, photosynthesis and WUE. Similarly, yield of fresh and dry weight and morphometric characteristics such as plant height, leaf area, aerial shoot length, and root length are greater as bocashi doses increased. A major finding of the present study is that oregano can perform better under shade-enclosure condition, the evidence also supports that bocashi doses and environment interacted to improve some variables depending on bocashi doses. It is recommended that complete or partial substitution of mineral fertilization can be carried out by using organic and bio-fertilizers which are safe and economic to farmers, at least for oregano plants. The interaction of environment with bocashi doses showed that physiological, yield and morphometric variables perform better as bocashi doses increased in both shade-enclosure and open-field conditions. We suggest that in future work the bocashi dose ranges need to be further broadened in order to find the optimum dose and be as inclusive as possible. Consequently, further investigations regarding soil physical, chemical, and mineral content related to fertility in oregano plants could be an important and interesting area of study. Results also suggest that future research should focus more on oregano plants under shade-enclosure, including of course other oregano species ecotypes or varieties. The results of the conducted experiments could have practical value in the future for evaluating other commercial herbs under different conditions and using bio fertilizers such as bocashi. This study can be used for better management of bocashi doses with oregano, which in turn can be used for cost-effective applications of bio fertilizers thereby leading to higher yield.

## Author Contributions

Conceived and designed the experiments: LM-P, BM-A. Performed the experiments: LM-P, ET-D, AN-G, and LH-M. Analysis of results was carried by: BM-A and ER-P. The following authors contributed providing funding for publication costs, materials and analysis tools: AN-G, LH-M, ET-D, and ER-P. Wrote, edited and revised the paper: BM-A, MC-M and ER-P. Approved the final version of the manuscript to be published: BM-A, LM-P, LH-M, ET-D, MC-M, ER-P and AN-G. All authors are in agreement that BM-A to be accountable for all aspects of the work in ensuring that questions related to the accuracy or integrity of any part of the work are appropriately investigated and resolved.

## Conflict of Interest Statement

The authors declare that the research was conducted in the absence of any commercial or financial relationships that could be construed as a potential conflict of interest.
